# Development and Validation of the Psychometric Properties of the FitMIND Foundation Sweets Addiction Scale—A Pilot Study

**DOI:** 10.3390/nu17121985

**Published:** 2025-06-12

**Authors:** Mikołaj Choroszyński, Joanna Michalina Jurek, Sylwia Mizia, Kamil Hudaszek, Helena Clavero-Mestres, Teresa Auguet, Agnieszka Siennicka

**Affiliations:** 1 Food Addiction Research Department, FitMIND Foundation, 03-435 Warsaw, Poland; 2 Grup de Recerca GEMMAIR (AGAUR)—Medicina Aplicada (URV), Departament de Medicina i Cirurgia, Universitat Rovira i Virgili (URV), Institut d’Investigació Sanitària Pere Virgili (IISPV), Mallafré Guasch, 4, 43007 Tarragona, Spain; joanna.michalina.jurek@gmail.com (J.M.J.);; 3 Division of Healthcare Innovations, Department of Public Health, Faculty of Health Sciences, Wroclaw Medical University, 50-367 Wroclaw, Polandagnieszka.siennicka@umw.edu.pl (A.S.); 4 Wroclaw University of Economics and Business, ul. Komandorska 118/120, 53-345 Wroclaw, Poland; 5 Servei Medicina Interna, Hospital Universitari de Tarragona Joan XXIII, Mallafré Guasch, 4, 43007 Tarragona, Spain

**Keywords:** food addiction, yale food addiction scale, psychometrics, emotional eating, obesity, ultra-processed foods

## Abstract

Background: The rising consumption of ultra-processed foods, especially those high in added sugars, poses a growing public health concern. Although several tools exist to assess food addiction, there is a lack of validated instruments specifically designed to measure addiction-like behaviors related to sweet food intake. Objectives: This study evaluates the psychometric properties of the FitMIND Foundation Sweets Addiction Scale (FFSAS), adapted from the Yale Food Addiction Scale 2.0 (YFAS 2.0), using data from Polish adults recruited through the FitMIND Foundation. Methods: The FFSAS was evaluated by 11 expert judges on four criteria: clarity, content validity, linguistic appropriateness, and construct representativeness. Afterwards, 344 adult volunteers (mean age 40.6 ± 10.7 years, 78% female, mean body mass index (BMI) 27.86 kg/m^2^) completed online FFSAS and provided demographic data, BMI, and self-reported sweets consumption. Internal consistency was assessed with Cronbach’s alpha and external validity was examined through Spearman’s correlations. Moreover, we conducted Exploratory and Confirmatory Factor Analyses (EFA and CFA). Results: Content validity of the FFSAS was supported by expert validation. The scale demonstrated good overall internal consistency (α = 0.85), with specific criteria such as tolerance (α = 0.916) and withdrawal (α = 0.914) showing particularly high reliability. The FFSAS total score was moderately correlated with sweets consumption frequency (ρ = 0.39, *p* < 0.05) and feelings of guilt (ρ = 0.35, *p* < 0.05). Exploratory factor analysis (EFA) revealed a robust three-factor structure, explaining 68.6% of the variance; the individual factors (subscales) derived from this structure demonstrated excellent internal consistency (Cronbach’s α ranging from 0.951 to 0.962). Sampling adequacy was high based on Kaiser–Meyer–Olkin measure (KMO = 0.956). Confirmatory factor analysis (CFA) indicated suboptimal model fit (Comparative Fit Index (CFI) = 0.74, Tucker–Lewis Index (TLI) = 0.69, Root Mean Square Error of Approximation (RMSEA) = 0.14), with a significant chi-square test (χ^2^ = 3761.76, *p* < 0.001). Conclusions: This pilot study demonstrated that the FFSAS may be a promising tool for assessing sweet food addiction in adults. Future research should focus on assessing the FFSAS’ suitability on more diverse populations in other countries for further validation.

## 1. Introduction

The Polish population has undergone significant shifts in dietary habits in recent years, marked by an increased consumption of ultra-processed foods (UPFs) and added sugars. This study aims to evaluate the prevalence and impact of sweets addiction within this population to inform public health interventions and clinical practice.

Food addiction, particularly to sweets, has garnered substantial research attention due to its strong association with metabolic diseases. The rising global prevalence of conditions such as obesity and type 2 diabetes mellitus (T2DM) represents a major public health challenge, contributing to increased mortality and disability-adjusted life years across all socioeconomic groups [1]. Among key modifiable risk factors, dietary behavior, such as the habitual consumption of UPFs, has been identified as a primary driver of these conditions [2,3]. UPFs, characterized by high levels of added sugars, refined carbohydrates, and saturated fats and low levels of fiber and micronutrients, are strongly linked to weight gain, systemic inflammation, insulin resistance, and impaired satiety signaling [4,5,6].

While traditional dietary patterns such as the Mediterranean diet are linked to improved metabolic outcomes and reduced chronic disease risk [7,8], the Western-style diet high in UPFs has been implicated in the development of metabolic disorders, including metabolic dysfunction-associated steatotic liver disease (MASLD), T2DM, cardiovascular complications [9,10], and dementia and Alzheimer’s disease [9,10,11]. In particular, excessive intake of sugar-sweetened foods and beverages, such as those high in fructose, can promote hepatic fat accumulation, mitochondrial dysfunction, and fibrosis [12,13].

Beyond metabolic outcomes, growing evidence suggests that UPFs, particularly those rich in added sugars, may exert addictive-like effects by stimulating reward pathways in the brain, leading to compulsive eating behaviors that mirror substance use disorders [14,15,16]. High consumption of sweet UPFs has also been linked to impaired cognitive function, mood instability, and increased risk of depression and anxiety, which may be mediated by blood glucose fluctuations, gut microbiota dysbiosis, and neuroinflammation [16,17,18,19]. Moreover, habitual intake of these foods can disrupt sleep quality and circadian rhythms, further aggravating metabolic and mental health issues [20]. In particular, in children and adolescents, excessive sugar intake has been associated with reduced academic performance, attention deficits, and increased hyperactivity [21]. These neurobiological and behavioral effects carry serious public health implications, especially in countries like Poland, where average sugar consumption far exceeds recommended levels, contributing to the burden of both physical and mental health conditions [22].

The FitMIND Foundation, established in 2022, is an organization, in which a group of experts in dietetics and psychology work in order to support patients struggling with a broad spectrum of eating disorders and related unhealthy behaviors. Despite its short history, the foundation has provided help and professional support to hundreds of patients from all over Poland. Practical observations from this experiences support the conclusion that there is a need for a tool to quantitatively assess the severity of sweets addiction, which is often a significant component of eating disorders.

In sum, we believe that there is a need for a validated, culturally adapted tool for evaluating problematic habits associated with sugary food consumption, which may be used for early detection of addictive-like behaviors towards sweet foods and inform public health strategies aimed at mitigating the metabolic and neuropsychological risks associated with excessive UPF intake.

Given the limitations of available tools to assess sweet food addiction, this pilot study aims to demonstrate the psychometric properties of the scale developed by the experts from the FitMIND Foundation (named the FitMIND Foundation Sweets Addiction Scale (FFSAS)), referring to the structure of the Yale Food Addiction Scale 2.0 (YFAS 2.0) based on external expert validation as well as extensive psychometric analysis.

## 2. Materials and Methods

### 2.1. Study Design

The validation of the FFSAS followed a two-phase design that combined expert judgement with psychometric testing.

#### 2.1.1. Phase I: Validation of External Experts

A panel of 11 expert judges was recruited to evaluate all 35 items of the FFSAS. The experts (mean experience = 16.5 years; range = 12–24) represented medicine (psychiatry), psychology, and health sciences. Each item was rated on a five-point Likert scale (1 = “strongly disagree”, 5 = “strongly agree”) across four dimensions: (i) clarity, (ii) content validity, (iii) linguistic appropriateness, and (iv) construct representativeness. For every item, the highest and lowest scores were discarded, and a mean of the remaining nine ratings was calculated and ranked; ties were resolved automatically by averaging the ranks and rounding to one decimal place. Items scoring < 3.5 were flagged for future revision (e.g., FFSAS9, 15, 30) [23]. Table 1 presents an overview of the recruited expert panel with details on field of expertise and specialization.

#### 2.1.2. Phase II: Psychometric Analysis

Psychometric analysis was based on data collected from participants and included the following: (a) internal consistency via Cronbach’s α; (b) construct validity via Spearman correlations between FFSAS scores and sweets-related variables; and (c) factorial validity via exploratory and confirmatory factor analyses (EFA, CFA).

### 2.2. Study Population

The study population involved in Phase II consisted of adult volunteers (aged ≥ 18 years), in accordance with Polish legal standards. The online questionnaire was distributed through the FitMIND Foundation’s network. The online survey was conducted from 13 January 2022 to 10 February 2025. No minimum sample size was predetermined; the aim was to collect data from as many participants as possible. The questionnaire included the FFSAS scale items, along with additional questions on demographics and eating behaviors, particularly related to sweets consumption. These questions are provided in the Supplementary Material (Table S1).

This study was approved by the Ethics Committee of Silesia Medical University (protocol code PCN/CBN/0022/KB/291/21, approved 1 January 2022) and conducted in accordance with the Declaration of Helsinki. All participants provided written informed consent, collected electronically via an online Google Forms questionnaire prior to participation.

### 2.3. Questionnaire Development

The FFSAS was developed by adapting the YFAS 2.0, a validated tool designed to assess food addiction based on the Diagnostic and Statistical Manual of Mental Disorders (DSM-5) criteria for substance use disorders [24]. The YFAS 2.0 comprises 35 items scored on an 8-point Likert scale (0 = “never”, 7 = “daily”), grouped into 11 addiction symptoms plus a 12th criterion assessing clinical distress or functional impairment. These criteria include the following: (1) consuming larger amounts of food or for longer than intended, (2) persistent desire or unsuccessful attempts to reduce consumption, (3) excessive time spent obtaining, using, or recovering from food, (4) neglect of important social, occupational, or recreational activities, (5) continued use despite knowledge of adverse consequences, (6) tolerance (increased amounts needed or reduced effect), (7) withdrawal symptoms or use to relieve them, (8) continued use despite social or interpersonal problems, (9) failure to fulfill major role obligations, (10) use in physically hazardous situations, (11) craving or strong urge to consume, and (12) distress or functional impairment caused by eating behavior. Each item has a predefined threshold (e.g., “once a month” to “4–6 times a week”) yielding a binary score (0 = not met, 1 = met). The total score, ranging from 0 to 11 (excluding the distress criterion for symptom count), determines food addiction severity: mild (2–3 symptoms), moderate (4–5 symptoms), or severe (≥6 symptoms), with diagnosis requiring the presence of distress [24].

For the FFSAS, the Polish version of YFAS 2.0, validated by Poprawa et al. (2020) [25] was used as the template, ensuring adequate translation. This adaptation retained the 35-item structure and 8-point Likert scale but replaced all references to “food” with “sweets” to focus exclusively on sweets-specific addiction-like behaviors.

The FFSAS assesses 12 criteria tailored to sweets consumption, including the following:

Loss of control e.g., “When I started eating sweets, I consumed much more than I planned” (FFSAS1).

Persistent desire or unsuccessful efforts to cut down e.g., “I really wanted to cut down or stop eating sweets but couldn’t” (FFSAS5).

Excessive time spent e.g., “When sweets were unavailable, I went out of my way to get them” (FFSAS10).

Neglect of activities e.g., “I ate sweets so often or in such quantities that I ate instead of working or spending time with family or friends” (FFSAS11).

Continued use despite negative consequences e.g., “I kept eating sweets despite knowing it harmed my health” (FFSAS29).

Tolerance e.g., “Over time, I had to eat more and more sweets to achieve satisfaction” (FFSAS18).

Withdrawal symptoms e.g., “When I cut down or stopped eating sweets, I felt irritable, nervous, or sad” (FFSAS19).

Overeating despite social problems e.g., “I had problems with family or friends due to overeating” (FFSAS24).

Failure to fulfill roles e.g., “My overeating interfered with caring for my family or doing household duties” (FFSAS27).

Use in hazardous situations e.g., “I was so distracted by eating sweets that I could have had an accident” (FFSAS30).

Craving e.g., “I had such an intense craving for sweets that I had to eat them immediately” (FFSAS33).

Distress and functional impairment e.g., “I had serious life problems due to eating, affecting daily organization, work, school, friends, family, or health” (FFSAS35).

Each criterion is measured by 1–4 items (see Annex for full list), with scoring thresholds adapted from YFAS 2.0. A sweets addiction diagnosis requires meeting at least two of the 11 symptom criteria, plus the 12th criterion (distress/impairment), within the past 12 months. The total symptom count (0–11) classifies severity as follows: no sweets addiction (No SA, 0–1 symptom without distress), mild (2–3 symptoms), moderate (4–5 symptoms), or severe (≥6 symptoms), with distress required for all diagnoses. Participants’ responses classify them into four groups: No SA, Mild SA, Moderate SA, or Severe SA. Additionally, the questionnaire includes items assessing the frequency of emotional responses related to sweets consumption (e.g., remorse, guilt, shame, anger) rated on the same 8-point scale (0 = “never”, 7 = “several times a day”), providing supplementary data on psychological correlates of sweets addiction. After data collection, the modified items were externally reviewed by an expert panel of 11 specialists (see Section 2.4.2. to ensure cultural relevance, linguistic clarity, and alignment with the Polish population’s context.

### 2.4. Statistical Analysis

#### 2.4.1. Descriptive Statistics

All statistical procedures were performed in IBM SPSS Statistics v26 and STATISTICA v13. Two-tailed *p* < 0.05 indicated significance; where several pairwise tests were run, the Bonferroni correction adjusted α.

Continuous variables (age, BMI, FFSAS scores) are presented as mean ± SD for data that met the Shapiro–Wilk normality criterion (*p* > 0.05) or as median [IQR] otherwise. Categorical variables (sex, education, place of residence) are shown as counts and percentages.

#### 2.4.2. Phase I—Expert-Rating Statistics

Eleven experts (mean experience = 16.5 years) rated each of the 35 FFSAS items on a 5-point Likert scale across four dimensions (clarity, content validity, linguistic appropriateness, construct representativeness) [23]. The highest and lowest score per item were discarded; trimmed means, SD and ranks were calculated in Excel. Items with a trimmed mean < 3.5 were flagged for revision; ties were resolved arithmetically by averaging consecutive ranks (no manual intervention).

#### 2.4.3. Reliability

Internal consistency was assessed with Cronbach’s α for each of the 12 DSM-5 criteria and for the total FFSAS score (35 items, 8-point scale).

#### 2.4.4. Construct Validity

Spearman’s rank-order correlations examined relationships between FFSAS scores (total and criteria) and sweets-related variables: frequency of sweets intake, negative emotions (remorse, guilt, shame, anger), and BMI.

#### 2.4.5. Group Comparisons

Independent t-tests compared total FFSAS scores across four BMI categories—underweight (<18.5 kg m^−2^), normal (18.5–24.9), overweight (25–29.9), and obese (≥30). Bonferroni-adjusted *p* values are reported.

#### 2.4.6. Factorial Validity

Exploratory factor analysis (EFA) used principal axis factoring with promax rotation; the number of factors was guided by eigenvalues > 1 and the scree-plot elbow. Sampling adequacy was confirmed with the Kaiser–Meyer–Olkin statistic (>0.80) and Bartlett’s test of sphericity (*p* < 0.001). The confirmatory factor analysis (CFA) evaluated the hypothesized three-factor model. Model fit was judged acceptable when CFI and TLI > 0.90, RMSEA < 0.08, and χ^2^/*df* < 3.0. The missing data were handled by pairwise deletion for descriptive analyses and listwise deletion for multivariate tests.

## 3. Results

The results are presented in the sections below, which were divided into two phases, Phase 1, presenting the outcomes from the FFSAS validation by the panel of expert judges, followed by Phase 2, showing the workflow of the study along with responses provided on the FFSAS questionnaire in the pilot with volunteering participants. The presented sample sizes in these samples may differ, which is the result of applying a pair-wise deletion to missing data. Consequently, the effective N differs across analyses and has been detailed in each table and figure.

### 3.1. Phase I: The FFSAS Validation Results by Panel of Expert Judges

The first phase of the presented study analysis was focused on the expert validation collected through the FFSAS data on behaviors related to food consumption, along with their perception of feelings associated with sugary foods intakes. The evaluation investigated these information by applying the assessment of different criteria.

For clarity, item scores ranged from 3.33 (FFSAS15, SD = 1.12) to 4.78 (FFSAS4, FFSAS32, SD < 0.47), with FFSAS4 and FFSAS19 scoring particularly high (>4.67) and exhibiting low variability. However, FFSAS15 and FFSAS30 scored below 3.5, indicating potential comprehension issues. In terms of content validity, scores ranged from 3.33 (FFSAS28, SD = 1.03) to 4.56 (FFSAS4, FFSAS19, SD < 0.69), with FFSAS9 and FFSAS30 being the lowest-scoring items. Regarding other parameters, linguistic appropriateness scores varied from 3.22 (FFSAS21, SD = 1.47) to 4.78 (FFSAS32, SD = 0.47) and construct representativeness ranged from 3.22 (FFSAS30, SD = 1.10) to 4.67 (FFSAS19, SD = 0.50). Items consistently scoring below 3.5, such as FFSAS9, FFSAS15, and FFSAS30, were flagged for revision in future iterations of the scale. All items from the FFSAS questionnaire can be found in the Supplementary Material (Table S1).

### 3.2. Phase II: Workflow and Characteristics of Study Participants

**Figure 1:**
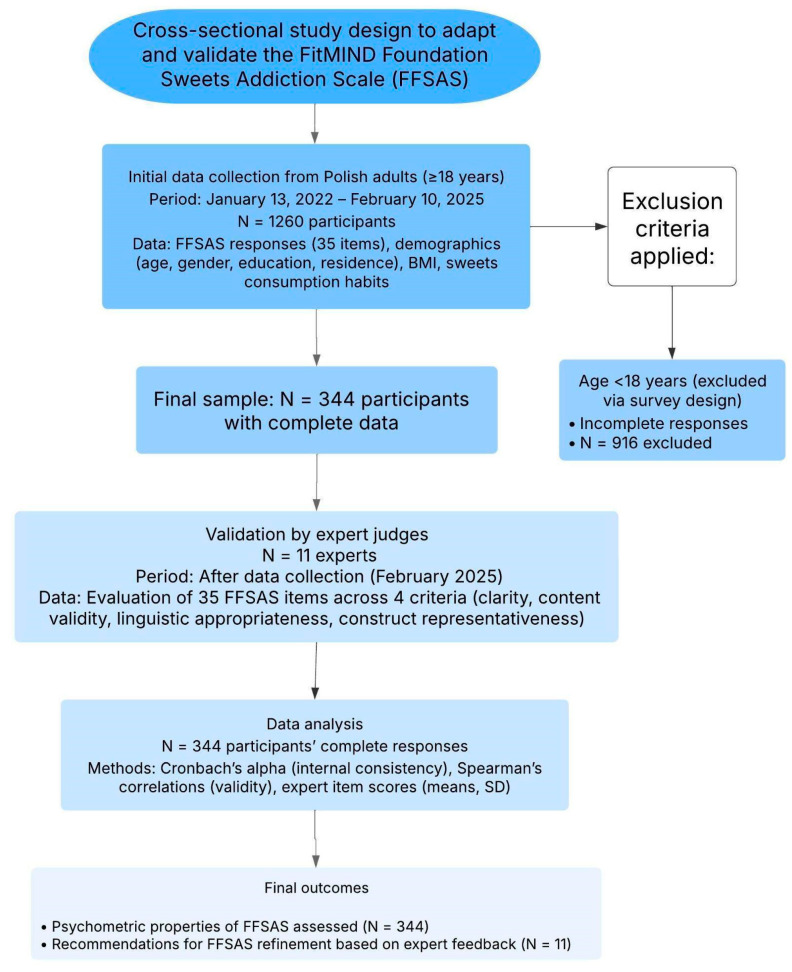
Workflow of the study methodology for the development and validation of the FFS.

Between 13 January 2022, and 10 February 2025, a total of 1260 Polish adults (aged ≥ 18 years) volunteered to participate in the study by completing the online FFSAS questionnaire via Google Forms. Of these, 916 participants were excluded due to incomplete responses (e.g., missing FFSAS items or demographic data). The study workflow along with the process of participant recruitment is demonstrated in Figure 1.

The final sample consisted of 344 participants with complete data on demographics, BMI, and sweets consumption habits, which was used for further analysis. This sample size met the requirements for factor analysis, as power calculations estimated a minimum sample size of 100–250 participants [26].

In this study, a total group of 344 Polish adults, including 270 (78%) women, and a mean age of 40.6 years (range 18–76) voluntarily responded online to the items of the developed FFSAS questionnaire. The demographic and anthropometric characteristics along with sub-group classification of participants are presented in Table 2.

Briefly, most of the participants in this study had higher education (72%) and resided in large cities with more than 500,000 inhabitants (34%). In this cohort, the mean BMI was 27.86 kg/m^2^, and 97 people (31%) of responders were classified as obese (BMI ≥ 30 kg/m^2^).

#### 3.2.1. Self-Reported Responses on Sweets Consumption and Addiction-like Behaviors

To assess the self-reported behaviors and emotional responses related to sweets consumption, participants responded to items in the FFSAS questionnaire concerning their identification as sweets addicts, admission of addiction, frequency and quantity of sweets intake, cravings or unsuccessful attempts to reduce consumption, and associated feelings of guilt or remorse. These data provide insight into the prevalence and patterns of addiction-like behaviors toward sweets in the total sample (*N* = 344). Results are summarized in Table 3.

In this study, most participants perceived their sweets consumption as problematic, with frequent intake and large quantities commonly reported, in contrast to self-reported addiction-like symptoms (e.g., cravings) and emotional distress (e.g., guilt). More than half of responders (62%) identified themselves as addicted to sweets when asked, “Do you identify as addicted to sweets?” while 82 (24%) did not, and 50 (15%) were uncertain (“Don’t know”). When asked directly about admitting addiction (e.g., to themselves or others), 183 (53%) confirmed it, 96 (28%) denied it, 29 (8%) were unsure, and 36 (10%) deemed the question not applicable. In this study, the frequency of sweets consumption was displayed as follows: 107 (31%) reported eating sweets several times a day, 64 (19%) once a day, 65 (19%) 3–4 times a week, 37 (11%) 5–6 times a week, 42 (12%) 1–2 times a week, and 29 (8%) less than once a week. In terms of quantity of consumed sweets, 173 participants (50.3%) reported consuming large amounts, and 170 participants (49.4%) reported small amounts.

In contrast, the self-reported behavioral indicators of addiction, such as cravings or unsuccessful attempts to quit, were less frequent in the study, with 29 (8%) affirming such experiences and 42 (12%) denying them. The frequency of experiencing emotional states associated with sweets consumption, in particular feelings of guilt or remorse were varied between participants, as 38 (11%) experienced these feelings several times a day, 19 (6%) once a day, 7 (2%) 5–6 times a week, 17 (5%) 3–4 times a week, 21 (6%) 1–2 times a week, 17 (5%) less than once a week, and 7 (2%) never, with 218 (63%) responses missing.

#### 3.2.2. Association Between Sweets Consumption, Sweets Addiction Severity, and Negative Emotions

**Figure 2:**
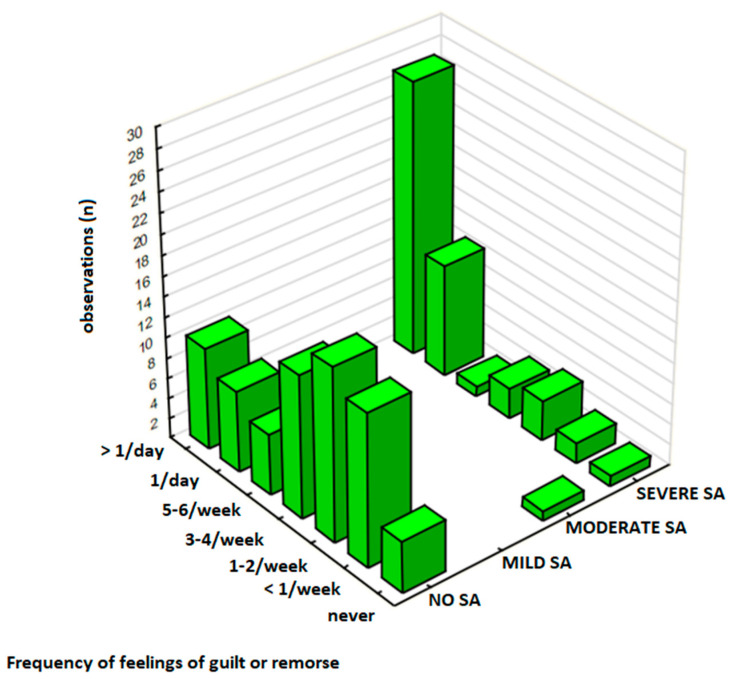
The frequency of contemplating sweets restriction by sweets addiction severity (SA) in the sub-sample (*N* = 126).

To further examine whether individuals with high sweets addiction (SA) severity experience greater emotional distress associated with sweet food intake, the relationship between sweets consumption frequency, SA severity, and negative emotions (e.g., guilt, remorse, shame, or anger) was assessed and results are presented in Table 2 (sub-sample, *N* = 126) and Table 3 (total sample, *N* = 344). For this analysis, the participants in the total sample were classified into four groups based on the overall FFSAS score: No SA (0–1 symptoms without distress), Mild SA (2–3 symptoms), Moderate SA (4–5 symptoms), and Severe SA (≥6 symptoms, with distress required for SA diagnosis, see Section 2.1.1). In this analysis, two key measures included sweets consumption frequency (Table 3, total sample, *N* = 344) and the frequency of negative emotions (Table 2, sub-sample *N* = 126), with results displayed in Figure 2.

#### 3.2.3. Sweets Consumption Frequency and SA Severity

Sweets consumption frequency was assessed in the total sample of 344 participants (Table 3), in which 62% (*n* = 213) were classified as No SA, 1% (*n* = 4) as Mild SA, 1% (*n* = 4) as Moderate SA, and 36% (*n* = 123) as Severe SA. Participants with Severe SA had significantly higher consumption rates of sweet foods, with 19% (*n* = 66) reporting eating sweets ‘several times a day’ compared to 12% (*n* = 40) in the No SA group. In this group, there was a strong association between sweet food consumption frequency and SA severity (χ^2^ = 85.23, *df* = 15, *p* < 0.00001).

#### 3.2.4. Negative Emotions and SA Severity

The frequency of negative emotions was evaluated in a subgroup of 126 participants (Table 2), in which 60% (*n* = 75) were No SA, 1% (*n* = 1) Mild SA, 1% (*n* = 1) Moderate SA, and 39% (*n* = 49) Severe SA. Overall, 30% (*n* = 38) of participants in this group experienced negative emotions towards sweets consumption ‘several times a day,’ including 21% (*n* = 27) participants with the Severe SA group and 8% (*n* = 10) in the No SA group. Emotional distress was significantly associated with SA severity (χ^2^, *p* = 0.00001), particularly among those with Severe SA, as evidenced by a moderate-to-strong positive correlation (γ = 0.629, *p* < 0.001).

### 3.3. Association Between Sweets Consumption and Negative Emotions

Participants without SA (No SA) exhibited relatively stable levels of negative emotions across all consumption frequencies, with only 8% (*n* = 10 out of 75 in the *N* = 126 subgroup) reporting frequent emotional distress (‘several times a day’), even among those consuming sweets daily (10%, *n* = 36 out of 213 in the *N* = 344 sample). In contrast, individuals with SA, especially Severe SA, reported a significantly higher prevalence of negative emotions, with the strongest concentration observed among those consuming sweets ‘several times a day’. A strong association between frequent sweets intake and emotional distress was observed in this group (χ^2^, *p* < 0.00001), with 55% (*n* = 27 out of 49) experiencing negative emotions at the highest frequency.

### 3.4. Patterns of Sweets Restriction Contemplation and Resolution Among Individuals with Sweets Addiction

To assess the individual differences in responses to sweets restriction, frequency of contemplating sweets restriction, experiencing negative emotions, consumption patterns, and persistent desires or unsuccessful attempts to limit sweets consumption were compared between the SA and No SA groups based on the scores reported values on a 7-point scale (for frequencies of contemplation, negative emotions, and sweets consumption, 1 = never, 7 = several times a day: Table 4, Table 5 and Table 6) and binary response (for persistent desire or control attempts, Yes/No: Table 7) in respect to the following questions on the FFSAS questionnaire: (1) “How often do you experience periods of contemplating restricting sweets? Promise of improvement or resolution ‘from tomorrow,’ ‘from Monday’?” (contemplation and resolutions) (Table 4), (2) “How often do you experience remorse, guilt, shame, or anger at yourself related to this?” (negative emotions) (Table 5), (3) “How often do you eat sweets?” (consumption frequency) (Table 6), and (4) “Is there a persistent desire or unsuccessful attempts to stop or control eating sweets?” (persistent desire/control attempts) (Table 7).

The differences in sample sizes across Table 4, Table 5, Table 6 and Table 7 reflect varying levels of response completeness for specific questionnaire items. Table 4 and Table 5 were analyzed in a subgroup of 126 participants who provided full responses to the variables included in these analyses. Table 6, however, was assessed using the total sample of 344 participants, as all participants responded to the relevant items. Table 7 was evaluated in a different subgroup of 166 participants who completed all items required for that specific analysis. These variations are due to partial missing data and were handled by conducting each analysis only on participants with complete data for the relevant variables. We recognize that these differences in sample size may be confusing and have now clarified them in the manuscript to ensure transparency and reproducibility.

#### 3.4.1. Contemplation, Resolutions, and Negative Emotions

The frequency of contemplating sweets restriction and making resolutions was analyzed in the subgroup of the sample (*N* = 126), and the results are presented in Table 4. The results show that 60% (*n* = 75) participants had No SA, 1% (*n* = 1) had Mild SA, 1% (*n* = 1) had Moderate SA, and 39% (*n* = 49) had Severe SA. Notably, 30% of respondents experienced negative emotions ‘several times a day,’ predominantly among those with Severe SA (21%, *n* = 27). The Severe SA group reported the highest frequency of contemplating sweet restriction, with 16% (*n* = 20) doing this ‘several times a day,’ compared to 10% (*n* = 12) in the No SA group. In this group, the contemplation frequency was positively correlated with SA severity (*p* < 0.001).

**Figure 3:**
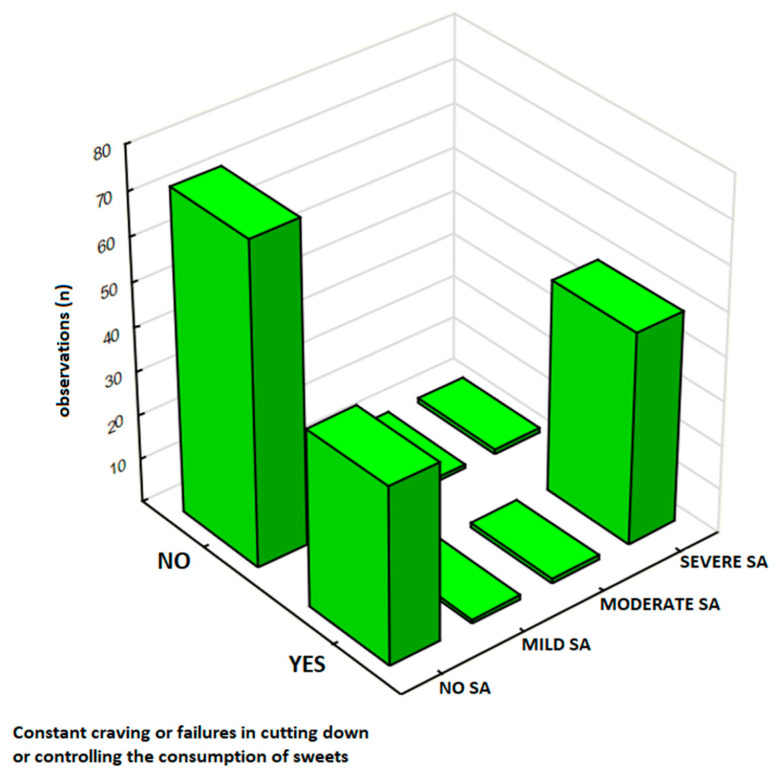
The frequency of experiencing negative emotions by sweets addiction severity in the sub-sample (*N* = 126).

Similarly, the frequency of negative emotions was significantly higher in participants with SA compared to those with No SA (Table 5). In this subgroup (*N* = 126), 30% (*n* = 38) responders experienced feelings of remorse, guilt, shame, or anger ‘several times a day,’ including 21% (*n* = 27) in the Severe SA group and 8% (*n* = 10) in the No SA group (Figure 3). Statistical analysis revealed a strong association between SA severity and the frequency of experiencing negative emotions (*p* = 0.00001) and a moderate-to-strong correlation between these variables (*p* < 0.001).

#### 3.4.2. The Frequency of Sweet Food Consumption

**Figure 4:**
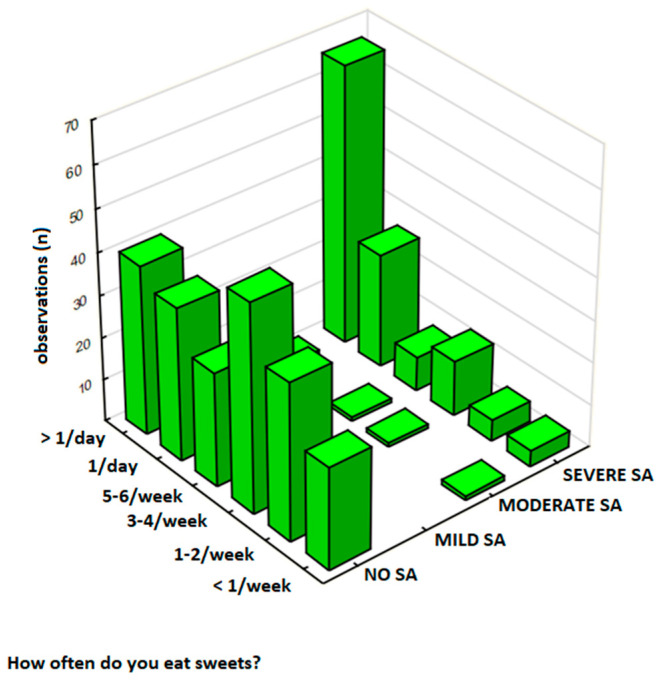
The frequency of sweet food consumption by sweets addiction severity in the total sample (*N* = 344).

The frequency of sweet food consumption was analyzed in a total sample of 344 participants, and results are presented in Table 6. In this group, 62% (*n* = 213) of respondents were classified as No SA, 1% (*n* = 4) as Mild SA, 1% (*n* = 4) as Moderate SA, and 36% (*n* = 123) as Severe SA. Participants with Severe SA had significantly higher consumption rates, with 19% (*n* = 66) eating sweets ‘several times a day’ compared to 12% (*n* = 40) in the No SA group. Notably, 31% of respondents reported sweet food intake ‘several times a day,’ with this behavior most frequent among those with Severe SA (19%, *n* = 66) compared to No SA (12%, *n* = 40) (Figure 4). In this analysis, there was a strong relationship between SA severity and the frequency of sweets consumption (*p* < 0.00001) and a moderate positive correlation between these variables (γ = 0.544, *p* < 0.001).

#### 3.4.3. The Persistent Desire and Control Attempts

**Figure 5:**
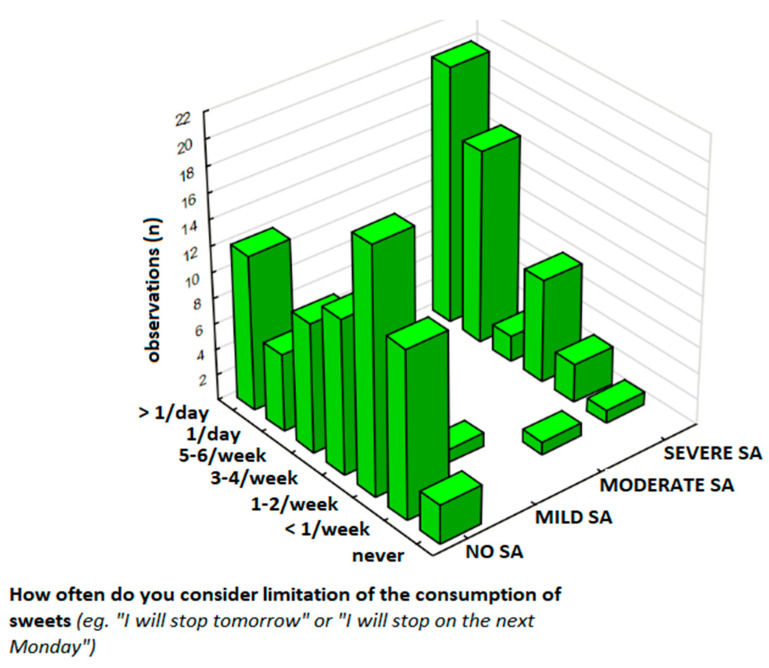
Persistent desire or unsuccessful attempts to control sweet food consumption in participants without sweets addiction (No SA) and participants with any degree of sweets addiction (SA) in the sub-sample (*N* = 166).

The persistent desire and unsuccessful attempts to control sweet food consumption was analyzed in a sub-sample of 166 respondents, and results are presented in Table 7. Participants were classified by SA severity: 68% (*n* = 113) as No SA, 1% (*n* = 2) as Mild SA, 1% (*n* = 2) as Moderate SA, and 30% (*n* = 49) as Severe SA. The results show that 54% (*n* = 90) reported ‘Yes,’ including 29% (*n* = 48) in the Severe SA group compared to 24% (*n* = 40) in the No SA group. Notably, 54% (*n* = 90) of respondents in this study reported experiencing a persistent desire or unsuccessful attempts to control sweets intake, with this response markedly higher among those with Severe SA (29%, *n* = 48) compared to No SA (24%, *n* = 40) (Figure 5). In this analysis, there was a significant association between SA severity and the presence of persistent desire or unsuccessful attempts to control eating sweets (*p* < 0.00001).

### 3.5. Assessment of the FFSAS Reliability and Internal Consistency

To assess the internal consistency of the FFSAS based on the preliminary data collected from the Polish population, Cronbach’s alpha (α) was computed for each of the 12 criteria evaluated by the questionnaire (Table 8). Overall, the FFSAS demonstrated strong internal consistency and reliability, with the overall α = 0.85; nevertheless, these values varied across different subscales, from 0.43 to 0.92. The high internal consistency was reported for Criterion 3, ‘Excessive Time Spent’, which assess the amount of time dedicated to obtaining, consuming, or thinking about sweets, demonstrating particularly high reliability (α = 0.916). This criterion was assessed through items such as “Spędziłem/am dużo czasu na jedzeniu przez cały dzień” (FFSAS9) and “Kiedy słodycze były niedostępne, byłem/am w stanie zrobić wiele, aby je zdobyć” (FFSAS10). Similarly, Criterion 7, ‘Withdrawal Symptoms’, evaluating the physical and emotional symptoms experienced after reducing sweets intake, showed strong internal consistency (α = 0.914). This subscale included five items such as “Kiedy ograniczałem/am lub zaprzestałem/am jedzenia słodyczy, czułem/am się poirytowany/a, nerwowy/a lub smutny/a” (FFSAS19) and “Kiedy ograniczałem/am lub zaprzestałem/am jedzenia słodyczy, miałem/am dolegliwości fizyczne” (FFSAS22).

In contrast, Criterion 10, ‘Use in Hazardous Situations’, determining whether participants consume or think about sweets in contexts that could lead to physical harm, showed lower internal consistency (α = 0.430). Items like “Byłem/am tak rozkojarzony/a przez spożywanie jedzenia, że mógłbym/mogłabym ulec wypadkowi” (FFSAS30) and “Byłem/am tak rozkojarzony/a przez myślenie o jedzeniu, że mógłbym/mogłabym ulec wypadkowi” (FFSAS31) yielded a weak alpha value, suggesting that these items may not consistently reflect hazardous behavior in this population. Also, Criterion 11, ‘Craving’, measuring the intensity and frequency of overwhelming urges to consume sweets, demonstrated weak internal consistency (α = 0.520).

#### Correlation Between the FFSAS Scores and Behavioral and Emotional Indicators of Sweets Addiction

To further explore the associations between the obtained FFSAS scores and self-reported behavioral and emotional measures, a series of correlations were executed, and results are presented in Table 9. In this study, significant positive associations were reported between FFSAS scores and several self-reported behaviors and emotional responses related to sweets consumption. Specifically, FFSAS scores were moderately correlated with self-identified sweets addiction (ρ = 0.38, *p* < 0.05) and frequency of sweets consumption (ρ = 0.3919, *p* < 0.05). A similar pattern was observed with feelings of guilt or remorse (ρ = 0.3497, *p* < 0.05), as higher the FFSAS scores were associated with stronger emotional distress regarding sweets consumption. Furthermore, the number of days per week with sweets consumption was strongly correlated with FFSAS scores (*p* < 0.05).

### 3.6. Preliminary Validation and Factor Extraction of the FFSAS Questionnaire

**Figure 6:**
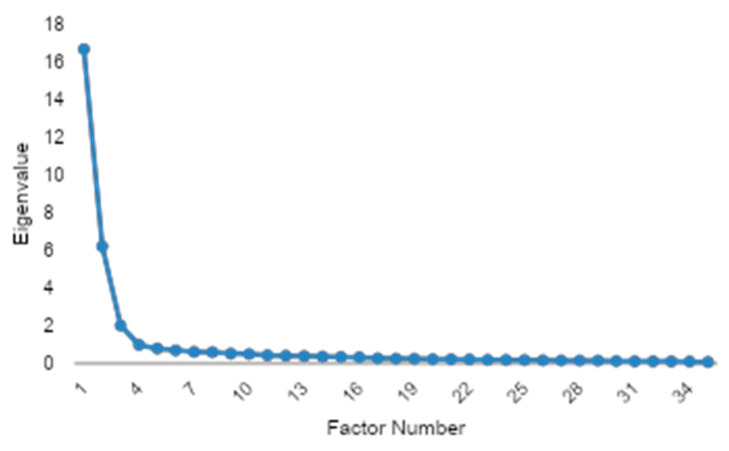
Based on the scree plot, a three-factor structure was identified.

To evaluate the structure of the FFSAS and ensure its psychometric properties, an EFA was conducted. Briefly, the applied Kaiser–Meyer–Olkin measure (KMO = 0.956) demonstrated excellent sampling adequacy, and Bartlett’s test of sphericity was highly significant (*p* < 0.001), confirming robust inter-item correlations and validating the dataset’s suitability for factor analysis. The number of factors was determined via the scree plot elbow criterion, which indicated a three-factor solution as the optimal point where eigenvalues began to level off sharply after the third component (Figure 6). The communality analysis revealed a high average communality of 0.686, with no variables below the 0.4 threshold and only one variable falling below 0.5, indicating that nearly all items were well-represented by the extracted factors.

The EFA indicated a robust three-factor structure, explaining 68.6% of the total variance. Thirty-seven percent of items exhibited cross-loadings, primarily between craving and loss-of-control constructs, suggesting nuanced interrelationships in sugar addiction phenotypes (Table 10). Each of the three extracted factors (subscales) demonstrated exceptional internal consistency (Cronbach’s α ranging from 0.951 to 0.962), though the high reliability coefficients and factor loadings above 0.90 for 18-item scales suggest potential item redundancy, necessitating future scale refinement to eliminate overlapping constructs while retaining psychometric robustness.

A CFA was subsequently conducted to validate the hypothesized factor structure, with the model explicitly defined according to the theoretical framework of the FFSAS, which operationalizes 12 diagnostic criteria. The CFA results demonstrated suboptimal fit (CFI = 0.74, TLI = 0.69, RMSEA = 0.14), prompting a potential reassessment of the FFSAS’s theoretical structure and necessitating item-level revisions to address cross-loadings and improve factorial validity. The statistically significant chi-square (χ^2^ = 3761.76, *p* < 0.001) confirmed model-data misfit, consistent with imperfect fit indices, necessitating post hoc model modifications to better align with the empirical covariance structure (Table 11). CFA revealed that, while several items loaded strongly onto their intended factors (e.g., FFSAS1 on CR01 and FFSAS5 on CR03 both with loadings of 1.0), some parameters exhibited atypically high values, such as FFSAS12 (loading = 63.44 on CR07) and FFSAS35 (loading = 21.46 on CR08), which may indicate estimation or model specification issues. A few items, including FFSAS9, demonstrated substantial cross-loadings, suggesting some overlap between latent constructs. Additionally, certain items, such as FFSAS33 (loading = 0.39 on CR10) and FFSAS34 (loading = 0.25 on CR10), showed relatively low factor loadings, which could reflect weaker associations with their respective factors. Despite these observations, the overall pattern of loadings provides valuable insights into the factorial structure of the scale and highlights areas for potential refinement. These results suggest that further review of specific items and possible model adjustments may enhance the psychometric properties and theoretical alignment of the instrument [27].

## 4. Discussion

The primary objective of this study was to evaluate the factor structure, psychometric properties, and external validation of the newly developed FFSAS, aimed at assessing sweets addiction. This scale, as an adaptation of the YFAS 2.0 dedicated to identifying addictive-like behaviors related to sweet food consumption in the general adult Polish population, demonstrated good overall reliability (α = 0.85). While the FFSAS showed moderate correlations with self-reported frequency of sweets intake (ρ = 0.39) and feeling of guilt upon their consumption (ρ = 0.35), indicating its ability to identify alerted eating habits specifically linked to UPFs rich in added sugar, future studies should aim to establish stronger associations with more objective behavioral or clinical outcomes to further solidify its criterion validity. Results of this pilot study using FFSAS indicated that a higher frequency of contemplating restriction and making resolutions among participants with SA was associated with the cycle of addiction, supporting the validity of FFSAS. Furthermore, the validation identified items of the FFSAS (e.g., FFSAS9, FFSAS15, FFSAS30) for refinement due to weaker subscales (α < 0.60), suggesting that future iterations could enhance psychometric properties.

Exploratory factor analysis (EFA) revealed a robust three-factor structure, explaining 68.6% of the variance. The individual subscales derived from this EFA demonstrated excellent internal consistency, with Cronbach’s α values ranging from 0.951 to 0.962. However, 37% of items showed cross-loadings, particularly between craving and loss-of-control constructs, and some items (e.g., FFSAS35, communality = 0.43) exhibited low communality, indicating potential redundancy or weak factor representation. Subsequently, Confirmatory Factor Analysis (CFA) showed suboptimal model fit (CFI = 0.74, TLI = 0.69, RMSEA = 0.14, χ^2^ = 3761.76, *p* < 0.001). Some items displayed atypically high loadings (e.g., FFSAS12 = 63.44, FFSAS35 = 21.46) or low loadings (e.g., FFSAS33 = 0.39, FFSAS34 = 0.25), suggesting potential estimation issues or construct overlap. These findings highlight the need for item-level revisions to improve factorial validity and reduce redundancy, providing a foundation for refining the FFSAS in future studies.

Compared to prior studies, FFSAS aligns with YFAS 2.0 findings, where approximately 15–20% of community samples meet food addiction criteria, with higher rates in females and overweight individuals [24,28]. This study, comprising 78% females with a mean BMI of 27.86 kg/m^2^, reflects these demographics, and the 62% self-identification as sweets-addicted (Table 2) underscores the scale’s relevance for sugar-focused compulsive eating. While the FFSAS shows promise in capturing sweets-specific addiction behaviors, the variability in item clarity and construct representativeness aligns with challenges seen in validating other food addiction scales, resultant from the complexity of defining food addiction and the inconsistencies in scale performance across different populations [29]. A series of correlation analyses between FFSAS items unraveling moderate associations with self-reported sweet food consumption frequency is consistent with other studies which suggest that food addiction constructs may not directly translate to simple consumption measures [30]. In addition, significant associations between feeling of guilt and emotional distress related to sweet food intake reported in this study provide early evidence supporting the notion that psychological factors play a crucial role in addictive-like eating behaviors, linking emotional dysregulation to excessive food intake [31]. Unlike general food addiction tools, FFSAS’s specificity to UPFs rich in added sugar may detect problematic alerted eating behaviors missed by broader measures, consistent with research highlighting sugar-rich foods as key triggers of addictive-like eating and contributors to metabolic disturbances [32].

Although the exact mechanisms through which addictive-like behaviors arise from regular consumption of sweet food in large amounts under psychological triggers like negative emotional states and stress remain unclear, experimental evidence suggests that excessive sweet intake activates dopamine reward pathways, paralleling drug addiction [21]. Animal studies have shown that sugar bingeing leads to tolerance and withdrawal, with dopamine receptor downregulation resembling substance dependence [33]. Neuroimaging studies with human participants have supported this, indicating a possible role of reward responses to sweet cues in food-addicted individuals [24,34,35]. These findings collectively support the involvement of neurobiological pathways in certain types of dependence on sugary foods, including urgency of cravings and loss of control [34,35,36].

The FFSAS scale was created based on a combination of psychological theories and eating behavior research, making it essential to evaluate its validity and reliability in non-clinical settings to ensure suitability for general populations [29]. Given that sweets addiction is associated with various psychological and behavioral factors, including emotional eating, impulsivity, and compulsive consumption [30,31,37,38], it is crucial to examine how effectively the FFSAS reflects these constructs. Consequently, the second part of this study focused on validation, which confirmed the clarity, content validity, linguistic appropriateness, and construct representativeness of the FFSAS questionnaire. Items FFSAS4 and FFSAS19 received the highest clarity scores, indicating strong conceptual representation, while FFSAS15 and FFSAS30 were rated lower, suggesting a need for revision. Similar trends were observed in content validity and construct representativeness, with items such as FFSAS28 and FFSAS30 scoring lower, highlighting areas for improvement. These findings are consistent with previous food addiction research on tools like the mYFAS 2.0, which also showed variability in factor structures depending on the population and cultural context [39,40,41].

Furthermore, our results indicate that higher FFSAS scores were associated with emotional distress and impulsive eating behaviors, aligning with existing literature linking sweet food addiction to emotional dysregulation and stress [31,37,42]. This supports the scale’s utility in identifying individuals at risk for maladaptive eating patterns, such as compulsive consumption and loss of control over sweets intake [24]. Notably, items related to behavioral patterns received higher expert ratings than those addressing emotional implications, suggesting that the scale currently places greater emphasis on observable behaviors. This distinction mirrors findings in other research on behavioral versus emotional components of addiction [42,43]. Future development of the FFSAS should consider enhancing its capacity to capture both dimensions, thereby improving its diagnostic depth and applicability in diverse psychological contexts.

In summary, while the FFSAS shows promise as a tool for assessing sweets-specific addiction, particularly in overweight women, its structure and usability require further refinement. Future research should focus on validating the scale in larger, more diverse populations, including clinical groups (e.g., individuals with obesity or eating disorders), and employing longitudinal designs to assess its predictive validity. Clinically, the FFSAS could aid in identifying at-risk individuals, enabling tailored interventions such as psychodietetic counseling and behavioral therapy for sweets cravings, and ultimately contributing to improved patient outcomes and public health strategies.

## 5. Limitations

This study has several limitations that should be considered when interpreting the findings. Firstly, the sample was predominantly female (78%) and relatively limited in size, which may affect the generalizability of the findings to broader populations, including men. Secondly, participants were recruited online through the FitMIND Foundation, an organization focused on eating behaviors. This recruitment method likely attracted individuals already interested in their eating habits, potentially introducing selection bias and possibly inflating the observed correlations between FFSAS scores and self-reported behaviors like sweets consumption and guilt.

Furthermore, the reliance on self-reported data for sweets consumption, emotional responses, and anthropometric measures (BMI) might be subject to recall bias or social desirability bias. The expert panel validation, while valuable for assessing content validity, revealed variability in ratings for several items (e.g., FFSAS15 and FFSAS30), indicating that these may require revision to improve clarity and relevance. The suboptimal model fit observed in the confirmatory factor analysis (CFA) (CFI = 0.74, TLI = 0.69, RMSEA = 0.14) underscores the need for further item refinement and additional CFA in larger, more diverse samples to confirm the scale’s factorial structure and improve its construct validity. Future iterations should also include test–retest reliability testing.

The cross-sectional design of the study limits our ability to draw causal inferences. The focus solely on sweets excludes other food categories that might contribute to addiction-like eating behaviors. Lastly, while the FFSAS scale was externally validated by an expert panel, the sampling method for expert evaluations might also introduce a degree of selection bias, reflecting specific theoretical viewpoints. Future research should prioritize larger, more diverse samples, employ longitudinal designs to assess predictive validity, validate the FFSAS in clinical populations, and explore measurement invariance across different demographic groups.

## 6. Conclusions

In summary, this pilot study presents preliminary evidence supporting the potential validity and reliability of the FFSAS, a Polish adaptation of the YFAS 2.0 tailored to assess addiction-like behaviors specifically related to sweets consumption. The scale demonstrated good overall internal consistency for the total score and its factors, along with construct validity indicated by associations with self-reported patterns of excessive intake, intense cravings, and guilt. Its targeted focus on sweet foods offers a potential advantage over broader food addiction instruments, possibly enabling more precise identification of individuals particularly vulnerable to high-sugar dietary patterns. These findings position the FFSAS as a potentially valuable and contextually relevant tool for both research and early intervention in the field of eating psychology. Nevertheless, continued validation in larger, more diverse, and clinical populations, including assessment of measurement invariance and criterion validity with objective behavioral measures, will be essential to fully establish its utility across settings and to inform public health strategies addressing the growing burden of ultra-processed, sugar-rich food consumption.

## Figures and Tables

**Table 1:** Composition of the expert panel validating the FFSAS.

Degree	Field	Specialization	Experience (Years)
Prof.	Medicine	Psychiatry; eating, anxiety, and personality disorders	24
PhD	Psychology	Self-regulation; behavioral addictions; psychometrics	20
PhD	Psychology	Research; business training; coaching; change management	20
MSc	Psychology	Psychometrics; organizational psychology	20
PhD	Psychology	Mental health; EMDR; consumer behavior	20
Dr hab.	Health Sciences	Clinical dietetics; obesity; MAFLD; CKD	18
PhD	Medicine	Psychiatry; psychotherapy; suicidology	15
PhD	Psychology	Psychodietetics; eating disorders	15
Dr hab.	Health Sciences	Clinical dietetics; psychodietetics; eating disorders	14
PhD	Psychology	Behavioral addictions; research methodology; psychometrics; statistics; mindfulness	12.5
PhD	Health Sciences	Public health; e-health; m-health; data analysis	12

**Table 2:** Demographic and anthropometric characteristics of study participants (total sample *N* = 344).

Characteristics	*N*	Values
Age	344	40.6 ± 10.7 (18–76)
Gender		
Women	270	78%
Men	74	22%
Education		
Primary	1	0%
Vocational	9	3%
Secondary	68	20%
During studies	18	5%
Higher degree	248	72%
Place of Residence		
Village (up to 2000 residents)	45	13%
Town (2000 to 50,000 residents)	76	22%
City (50,000 to 250,000 residents)	72	21%
City (250,000 to 500,000 residents)	34	10%
City (over 500,000 residents)	117	34%
BMI (kg/m^2^)	336	27.86 ± 6.6
Underweight (<18.5)	8	2%
Normal weight (18.5–24.9)	111	32%
Overweight (25–29.9)	120	35%
Obese (≥30)	97	31%

Data is presented as numbers (*N*) and percentages (%). BMI (kg/m^2^) and age (years) are presented as mean ± SD. Participants were classified by BMI according to WHO criteria. BMI, Body Mass Index; SD, Standard Deviation.

**Table 3:** Reported sweet food consumption and addiction in the total sample (*N* = 344).

Criterion	Response	*N*	%
Sweets addiction self-identification:	Yes	212	62
	No	82	24
	Don’t know:	50	15
Admission of addiction:	Yes:	183	53
	No:	96	28
	Don’t know:	29	8
	Not applicable:	36	10
Frequency of sweets consumption:	Less than 1 time a week:	29	8
	1–2 times a week:	42	12
	3–4 times a week:	65	19
	5–6 times a week:	37	11
	Once a day:	64	19
	Several times a day:	107	31
Quantity of sweets consumed:	Small:	170	49
	Large:	173	50
	Missing data:	1	0
Craving or unsuccessful attempts to quit:	Yes:	29	8
	No:	42	12
	Missing data:	65	19
Frequency of sweets cravings:	Never:	3	1
	Less than 1 time a week:	15	4
	1–2 times a week:	23	7
	3–4 times a week:	20	6
	5–6 times a week:	12	3
	Once a day:	21	6
	Several times a day:	32	9
	Missing data:	218	63
Feelings of guilt or remorse:	Never:	7	2
	Less than 1 time a week:	17	5
	1–2 times a week:	21	6
	3–4 times a week:	17	5
	5–6 times a week:	7	2
	Once a day:	19	6
	Several times a day:	38	11
	Missing data:	218	63

Data is presented as numbers (*N*) and percentages (%) for the total sample (*N* = 344). Percentages may not sum to 100% due to rounding or missing data, which are indicated where applicable. ‘Sweets addiction self-identification’ reflects responses to the question ‘Do you identify as addicted to sweets?’, with ‘Don’t know’ responses included as a separate category.

**Table 4:** Comparison of the frequency of contemplating sweets restriction between participants without sweets addiction (No SA) and participants with any degree of sweets addiction (SA) in the sub-sample (*N* = 126).

How Often Do You Experience Periods of Contemplating Restriction? Promise of Improvement or Resolution “from Tomorrow”, “from Monday”?	Classification SA				
	No SA	Mild SA	Moderate SA	Severe SA	Total
Never	2 (3)	0 (0)	0 (0)	0 (0)	2 (3)
Less than once a week	10 (13)	0 (0)	1 (1)	1 (1)	12 (15)
1–2 times a week	15 (19)	1 (1)	0 (0)	2 (3)	18 (23)
3–4 times a week	10 (12)	0 (0)	0 (0)	6 (8)	16 (20)
5–6 times a week	8 (10)	0 (0)	0 (0)	2 (3)	10 (12)
Once a day	5 (6)	0 (0)	0 (0)	12 (15)	17 (21)
Several times a day	10 (12)	0 (0)	0 (0)	16 (20)	25 (32)
Total	60 (75)	1 (1)	1 (1)	39 (49)	100 (126)
χ^2^, *p* = 0.00046					
γ = 0.573, *p* < 0.001					

Data is presented as percentages and number counts (*n*) calculated relative to the subgroup total (*N* = 126). A chi-square test (χ^2^) was computed to assess the association between contemplation frequency and SA severity (*df* = 18, *p* = 0.00046). The Goodman–Kruskal gamma coefficient (γ) was employed as a measure of the strength and direction of this relationship between the frequency of contemplating sweets restriction and SA severity (γ = 0.573, *p* < 0.001). *p* < 0.05 considered as significant.

**Table 5:** Comparison of the frequency of experiencing negative emotions between participants without sweets addiction (No SA) and participants with any degree of sweets addiction (SA) in the sub-sample (*N* = 126).

How Often Do You Experience Remorse, Guilt, Shame, or Anger at Yourself Related to This?	Classification SA				
	No SA	Mild SA	Moderate SA	Severe SA	Total
Never	4 (5)	0 (0)	1 (1)	1 (1)	6 (7)
Less than once a week	12 (15)	0 (0)	0 (0)	2 (2)	13 (17)
1–2 times a week	13 (17)	0 (0)	0 (0)	3 (4)	17 (21)
3–4 times a week	11 (14)	0 (0)	0 (0)	2 (3)	13 (17)
5–6 times a week	5 (6)	0 (0)	0 (0)	1 (1)	6 (7)
Once a day	6 (8)	0 (0)	0 (0)	9 (11)	15 (19)
Several times a day	8 (10)	1 (1)	0 (0)	21 (27)	30 (38)
Total	60 (75)	1 (1)	1 (1)	39 (49)	100 (126)
χ^2^, *p* = 0.00001					
γ = 0.629, *p* < 0.001					

Data is presented as percentages and number counts (*n*) calculated relative to the subgroup total (*N* = 126). A chi-square test (χ^2^) was computed to assess the association between contemplation frequency and SA severity (*df* = 18, *p* = 0.00001). The Goodman–Kruskal gamma coefficient (γ) was employed as a measure of the strength and direction of this relationship between the frequency of contemplating sweets restriction and SA severity (γ = 0.629, *p* < 0.001). *p* < 0.05 considered as significant.

**Table 6:** Comparison of the frequency of the sweet food consumption between participants without sweets addiction (No SA) and participants with any degree of sweets addiction (SA) in the total sample (*N* = 344).

How Often Do You Eat Sweets?	Classification SA				
	No SA	Mild SA	Moderate SA	Severe SA	Total
Never	7 (24)	0 (0)	0 (0)	1 (4)	8 (29)
Less than once a week	11 (37)	0 (0)	0 (0)	1 (5)	12 (42)
1–2 times a week	14 (49)	1 (2)	0 (1)	4 (13)	19 (65)
3–4 times a week	8 (27)	0 (1)	0 (1)	2 (8)	11 (37)
5–6 times a week	10 (36)	0 (1)	0 (0)	8 (27)	19 (64)
Once a day	12 (40)	0 (0)	0 (1)	19 (66)	31 (107)
Several times a day	62 (213)	1 (4)	1 (4)	36 (123)	100 (344)
χ^2^, *p* < 0.00001					
γ = 0.544, *p* < 0.001					

Data is presented as percentages and number counts (*n*) calculated relative to the subgroup total (*N* = 126). A chi-square test (χ^2^) was computed to assess the association between contemplation frequency and SA severity (χ^2^ = 85.23, *df* = 15, *p* = 0.00001). The Goodman–Kruskal gamma coefficient (γ) was employed as a measure of the strength and direction of this relationship between the frequency of contemplating sweets restriction and SA severity (γ = 0.544, *p* < 0.001). *p* < 0.05 considered as significant.

**Table 7:** Reported persistent desire or unsuccessful attempts to control sweet food consumption in participants without sweets addiction (No SA) and participants with any degree of sweets addiction (SA) in the sub-sample (*N* = 166).

Is There a Persistent Desire or Unsuccessful Attempts to Stop or Control Eating Sweets?	Classification SA				
	No SA	Mild SA	Moderate SA	Severe SA	Total
Yes	24 (40)	1 (1)	1 (1)	29 (48)	54 (90)
No	44 (73)	1 (1)	1 (1)	1 (1)	46 (76)
Total	68 (113)	1 (2)	1 (2)	30 (49)	100 (166)
χ^2^, *p* < 0.00001					

Data is presented as percentages and number counts (*n*) calculated relative to the subgroup total (*N* = 166). A chi-square test (χ^2^) was computed to assess the association between contemplation frequency and SA severity (*df* = 3, *p* = 0.00001). The Goodman–Kruskal gamma coefficient (γ) was employed as a measure of the strength and direction of this relationship between the frequency of contemplating sweets restriction and SA severity (γ = 0.544, *p* < 0.001). *p* < 0.05 considered as significant.

**Table 8:** The computed Cronbach’s alpha (α) for all FFSAS criteria.

Criterion	Number of Items	Sum of Item Variances	Variance of Summed Items	Cronbach’s Alpha
1	3	13.68815682	32.31926741	0.864706044
2	4	25.82650221	75.678207	0.878310181
3	3	18.20527663	46.77292528	0.916159781
4	4	22.29518103	38.16421452	0.554412336
5	2	12.16105329	19.50280527	0.752891892
6	2	10.85493084	19.04643535	0.860161427
7	5	26.24855922	97.68957217	0.914133046
8	3	14.63365313	29.18580921	0.747905736
9	2	9.778273103	14.89375551	0.686929821
10	3	19.74027053	27.6627568	0.429593098
11	2	11.89428944	16.06928436	0.51962425
12	2	10.59159943	16.29869822	0.70031345

Cronbach’s alpha values (α) reflect internal consistency for each of the 12 DSM-5-based addiction criteria assessed by the FFSAS, calculated from data obtained from a total sample of (*N* = 344). ‘Items’ indicates the number of questions per criterion. Sum of item variances and variances of summed items are provided for transparency of reliability computations.

**Table 9:** The associations between FFSAS scores and self-reported behavioral and emotional measures of sweets consumption.

FFSAS Score	Variables	ρ	*p* Value
1	Self-identified sweets addiction	0.38	<0.05
2	Admission of addiction to loved ones	0.28	<0.05
3	Frequency of sweets consumption	0.39	<0.05
4	Quantity of sweets consumed	0.23	<0.05
5	Frequency of thoughts about limiting sweets	0.39	<0.05
6	Frequency of feelings of guilt or remorse	0.35	<0.05
7	Frequency of evening snacking	0.44	<0.05
8	Number of days per week with sweets consumption	0.48	<0.05

Variables reflect self-reported measures over the past 12 months, with missing data excluded pairwise from analyses. Spearman’s rank correlation coefficients (ρ) were calculated for a total sample of (*N* = 344). FFSAS scores represent the total number of met criteria (0–11). *p* values < 0.05 considered as significant.

**Table 10:** EFA rotated loadings matrix of EFA.

Factor 1	Factor 2	Factor 3	Communality	
YFAS 1	0.28	0.36	0.67	0.66
YFAS 2	0.30	0.34	0.71	0.72
YFAS 3	0.36	0.44	0.43	0.51
YFAS 4	0.29	0.40	0.76	0.83
YFAS 5	0.49	0.25	0.67	0.76
YFAS 6	0.70	0.03	0.53	0.77
YFAS 7	0.59	0.13	0.60	0.72
YFAS 8	0.78	0.06	0.16	0.64
YFAS 9	0.77	0.18	0.08	0.63
YFAS 10	0.79	−0.02	0.22	0.68
YFAS 11	0.17	0.79	0.19	0.70
YFAS 12	0.06	0.87	0.12	0.77
YFAS 13	0.18	0.85	0.17	0.78
YFAS 14	0.21	0.75	0.05	0.62
YFAS 15	0.32	0.59	0.50	0.69
YFAS 16	0.43	0.52	0.46	0.67
YFAS 17	0.71	0.29	0.18	0.62
YFAS 18	0.72	0.32	0.17	0.65
YFAS 19	0.83	0.06	0.24	0.74
YFAS 20	0.84	−0.01	0.29	0.79
YFAS 21	0.86	−0.05	0.25	0.81
YFAS 22	0.26	0.66	0.33	0.61
YFAS 23	0.54	0.27	0.58	0.69
YFAS 24	0.34	0.71	0.11	0.63
YFAS 25	−0.18	0.87	0.23	0.85
YFAS 26	0.30	0.68	0.17	0.58
YFAS 27	0.63	0.47	−0.03	0.63
YFAS 28	−0.17	0.88	0.21	0.84
YFAS 29	0.55	0.25	0.52	0.64
YFAS 30	−0.05	0.79	0.26	0.70
YFAS 31	−0.12	0.64	0.45	0.62
YFAS 32	0.17	0.61	0.54	0.69
YFAS 33	0.78	0.05	0.27	0.69
YFAS 34	0.78	0.10	0.16	0.65
YFAS 35	0.46	0.47	0.02	0.43

YFAS, Yale Food Addiction Scale. Rotated factor loadings are presented from an EFA using Varimax rotation. Loadings ≥ 0.40 are considered meaningful. The communality column represents the proportion of each item’s variance explained by the three extracted factors.

**Table 11:** CFA loadings matrix.

	CR01	CR02	CR03	CR04	CR05	CR06	CR07	CR08	CR09	CR10	CR11	CR12
YFAS1	1.00											
YFAS2	1.04											
YFAS3	0.86											
YFAS4		1.00										
YFAS5			1.00									
YFAS6			1.02									
YFAS7			1.00				1.00					
YFAS8				1.00								
YFAS9								1.00	1.00			
YFAS10				1.03								
YFAS11							61.85					
YFAS12							63.44					
YFAS13							64.35					
YFAS14							56.25					
YFAS15							52.44					
YFAS16												1.00
YFAS17												0.95
YFAS18				0.98								
YFAS19									1.17			
YFAS20				1.20								
YFAS21								45.32				
YFAS22					1.00							
YFAS23					1.18							
YFAS24						1.00						
YFAS25		1.10										
YFAS26						0.99						
YFAS27									0.94			
YFAS28										1.00		
YFAS29											1.00	
YFAS30											1.26	
YFAS31		1.05										
YFAS32		1.13										
YFAS33										0.39		
YFAS34										0.25		
YFAS35								21.46				

YFAS, Yale Food Addiction Scale. The Construct Reliability (CR) values calculated for items of the YFAS mapped onto latent factors (CR01–CR12). Values represent item contributions to the internal consistency of the constructs, with higher values indicating stronger reliability. CR values above 0.70 are considered acceptable. Blank cells indicate no significant loading of the item on the corresponding factor or that the item was not included in the construct reliability calculation for that factor. Rotated factor loadings are presented from an EFA using Varimax rotation. Loadings ≥ 0.40 are considered meaningful. The communality column represents the proportion of each item’s variance explained by the three extracted factors.

## Data Availability

The dataset used during the current study is available from the corresponding author on reasonable request.

## Supplementary Materials

The following supporting information can be downloaded at: https://www.mdpi.com/article/10.3390/nu17121985/s1. Table S1. The FitMIND Foundation Sweets Addiction Scale (FFSAS) Items.
