# Supplementary material for: Development and Validation of the Psychometric Properties of the FitMIND Foundation Sweets Addiction Scale—A Pilot Study
Source: Nutrients. 2025 Jun 12;17(12):1985. doi: 10.3390/nu17121985 (PMC12196253; doi:10.3390/nu17121985)
Supplement: Supplementary file 1 [file nutrients-17-01985-s001.zip › nutrients-3693625-supplementary.pdf]

Supplementary Material

Table S1. The FitMIND Foundation Sweets Addiction Scale (FFSAS) Items.

Diagnostic and Statistical Manual of Mental Disorders, Fifth Edition (DSM-5) Category	FFSAS Question Number	YFAS 2.0 Question text (English)	FFSAS Question text (Polish translation)
1. Substance taken in larger amount and for longer period than intended	FFSAS1	*When I started to eat certain foods, I ate much more than planned.*	*Kiedy zaczynałem jeść słodycze, jadłem o wiele więcej, niż planowałem.*
	FFSAS2	*I ate certain foods even though I was no longer hungry.*	*Jadłem słodycze, mimo że nie byłem już głodny.*
	FFSAS3	*I ate to the point where I felt physically ill.*	*Jadłem do momentu, aż czułem się fizycznie źle.*
2. Persistent desire or repeated unsuccessful attempts to quit	FFSAS4	*I worried a lot about cutting down on certain types of food, but I ate them anyways.*	*Martwiłem się o ograniczenie jedzenia słodyczy, ale i tak je jadłem.*
	FFSAS5	*I really wanted to cut down on or stop eating certain kinds of food, but I just couldn't.*	*Naprawdę chciałem ograniczyć lub przestać jeść słodycze, ale nie mogłem.*
	FFSAS6	*I tried to cut down on or not eat certain kinds of food, but I wasn't successful.*	*Próbowałem ograniczyć lub nie jeść słodyczy, ale mi się nie udało.*
	FFSAS7	*I tried and failed to cut down on or stop eating certain foods.*	*Próbowałem i nie udało mi się ograniczyć lub przestać jeść słodyczy.*
3. Much time/activity to obtain, use, recover	FFSAS8	*I spent a lot of time feeling sluggish or tired from overeating.*	*Spędzałem dużo czasu czując się ospały lub zmęczony z powodu przejedzenia słodyczami.*
	FFSAS9	*I spent a lot of time eating certain foods throughout the day.*	*Spędzałem dużo czasu jedząc słodycze przez cały dzień.*

	FFSAS10	*When certain foods were not available, I went out of my way to get them.*	*Gdy słodycze nie były dostępne, starałem się je zdobyć.*
4. Important social, occupational, or recreational activities given up or reduced	FFSAS11	*I ate certain foods so often or in such large amounts that I stopped doing other important things.*	*Jadłem słodycze tak często lub w takich ilościach, że przestałem robić inne ważne rzeczy.*
	FFSAS12	*I avoided work, school, or social activities because I was afraid I would overeat there.*	*Unikałem pracy, szkoły lub spotkań towarzyskich, bo bałem się, że tam się przejem słodyczami.*
	FFSAS13	*I felt so bad about overeating that I didn't do other important things.*	*Czułem się tak źle z przejedzenia słodyczami, że nie robiłem innych ważnych rzeczy.*
	FFSAS14	*I avoided work, school, or social functions because I could not eat certain foods there.*	*Unikałem pracy, szkoły lub spotkań, bo nie mogłem tam jeść słodyczy.*
5. Use continues despite knowledge of adverse consequences	FFSAS15	*I kept eating in the same way even though my eating caused emotional problems.*	*Jadłem słodycze w ten sam sposób, mimo że powodowało to u mnie problemy emocjonalne.*
	FFSAS16	*I kept eating in the same way even though my eating caused physical problems.*	*Jadłem słodycze w ten sam sposób, mimo że powodowało to u mnie problemy fizyczne.*
6. Tolerance (marked increase in amount; marked decrease in effect)	FFSAS17	*Eating the same amount of food did not give me as much enjoyment as it used to.*	*Jedzenie tej samej ilości słodyczy nie dawało mi już tyle przyjemności co kiedyś.*
	FFSAS18	*I needed to eat more and more to get the feelings I wanted from eating.*	*Musiałem jeść coraz więcej słodyczy, by poczuć to, co chciałem.*

7. Characteristic withdrawal symptoms; substance taken to relieve withdrawal	FFSAS19	*I felt anxious or nervous when I cut down or stopped eating certain foods.*	*Czułem się niespokojny lub nerwowy, gdy ograniczałem lub przestawałem jeść słodycze.*
	FFSAS20	*I felt tired or fatigued when I cut down or stopped eating certain foods.*	*Czułem się zmęczony, gdy ograniczałem lub przestawałem jeść słodycze.*
	FFSAS21	*I had trouble sleeping when I cut down or stopped eating certain foods.*	*Miałem problemy ze snem, gdy ograniczałem lub przestawałem jeść słodycze.*
	FFSAS22	*I felt irritable when I cut down or stopped eating certain foods.*	*Byłem rozdrażniony, gdy ograniczałem lub przestawałem jeść słodycze.*
	FFSAS23	*I ate certain foods to prevent feelings like anxiety or nervousness.*	*Jadłem słodycze, by uniknąć uczucia niepokoju lub nerwowości.*
8. Continued use despite social or interpersonal problems	FFSAS24	*Overeating caused problems with my family or friends.*	*Przejadanie się słodyczami powodowało problemy z moją rodziną lub przyjaciółmi.*
	FFSAS25	*Eating certain foods caused problems in my relationships with other people.*	*Jedzenie słodyczy powodowało problemy w moich relacjach z innymi ludźmi.*
	FFSAS26	*My overeating caused me significant problems with people who are important to me.*	*Moje przejadanie się słodyczami powodowało poważne problemy z ludźmi, którzy są dla mnie ważni.*
9. Failure to fulfill major role obligation (e.g., work, school, home)	FFSAS27	*My eating caused me to not meet my responsibilities at work, school, or home.*	*Moje jedzenie słodyczy sprawiało, że nie wypełniałem obowiązków w pracy, szkole lub domu.*

	FFSAS28	*I was too distracted by eating or thinking about food to get my work done.*	*Byłem zbyt rozproszony jedzeniem lub myśleniem o słodyczach, by wykonać swoją pracę.*
10. Use in physically hazardous situations	FFSAS29	*I ate certain foods even though it was dangerous (e.g., while driving).*	*Jadłem słodycze, mimo że to niebezpieczne (np. podczas jazdy).*
	FFSAS30	*I ate certain foods in situations where it could have been physically risky.*	*Jadłem słodycze w sytuacjach, które mogły być fizycznie ryzykowne.*
	FFSAS31	*Eating certain foods put me in physically dangerous situations.*	*Jedzenie słodyczy stawiało mnie w fizycznie niebezpiecznych sytuacjach.*
11. Craving, or a strong desire or urge to use	FFSAS32	*I had strong urges to eat certain foods that were hard to control.*	*Miałem silną, trudną do opanowania chęć jedzenia słodyczy.*
	FFSAS33	*I had intense cravings for certain foods.*	*Miałem intensywne pragnienie jedzenia słodyczy.*
12. Use causes clinically significant impairment or distress	FFSAS34	*My eating habits caused me significant distress.*	*Moje nawyki jedzenia słodyczy powodowały u mnie znaczny niepokój.*
	FFSAS35	*My eating habits caused me significant problems in my ability to function.*	*Moje nawyki jedzenia słodyczy powodowały poważne problemy w moim funkcjonowaniu.*
